# miR-503 Is Involved in the Protective Effect of Phase II Enzyme Inducer (CPDT) in Diabetic Cardiomyopathy via Nrf2/ARE Signaling Pathway

**DOI:** 10.1155/2017/9167450

**Published:** 2017-12-18

**Authors:** Ying Miao, Qin Wan, Xiaoyu Liu, Yu Wang, Yi Luo, Dan Liu, Nengbo Lin, Honggang Zhou, Jiyuan Zhong

**Affiliations:** Department of Endocrinology, The Affiliated Hospital of Southwest Medical University, Luzhou 646000, China

## Abstract

Diabetic cardiomyopathy (DCM) is a common heart disease. The Phase II enzyme inducer (CPDT) is a complex enzyme that promotes the expression of antioxidant enzymes through activating nuclear factor erythroid 2-related factor 2 (Nrf2); these compounds have been shown to protect against oxidative stress. However, whether these compounds have similar protective effects in DCM still remains unclear. The purpose of this study is to investigate the protective effects and potential mechanism of CPDT in diabetic cardiomyopathy. In the results, firstly, compared with control rats, myocardial cell size, left ventricular mass index, and myocardial apoptosis index were increased, miR-503 was increased, and Nrf2, malondialdehyde (MDA), and heme oxygenase 1 (HO-1) were decreased in diabetic cardiomyopathy rats. Furthermore, compared with diabetic cardiomyopathy rats, these above parameters show the opposite change in CPDT treatment rats. In addition, the bioinformatics and luciferase reporter assay demonstrated that Nrf2 is a direct target of miR-503. Finally, the miR-503 could also regulate Nrf2 in the myocardial cells. Therefore, miR-503 is involved in the protective effect of CPDT in diabetic cardiomyopathy via Nrf2/ARE signaling pathway; miR-503 and Nrf2 may be a promising therapeutic target for the management of diabetic cardiomyopathy.

## 1. Background

Diabetic cardiomyopathy (DCM) is the main cardiovascular complication, which occurs in approximately 60% of patients with well-controlled diabetes, resulting in systolic dysfunction with diastolic dysfunction, which is an independent risk for any vascular disease or hypertension [1]. An increasing body of evidences implicate hyperglycemia, lipotoxicity, and mitochondrial uncoupling in contributing to cardiac inflammation, which play an important role in the pathogenesis and progression of diabetic cardiomyopathy [2, 3]. In this process, cytoplasmic calcium was increased, and then, triggering mitochondrial changes, the production of reactive oxygen species (ROS) was increased, and activating ROS levels leads to oxidative damage in diabetic cardiomyopathy [4].

Phase II enzyme inducer such as 5,6-dihydrocyclopenta-1,2-dithiole-3-thione (CPDT), 3H-1,2-dithiole-3-thione (D3T), and tert-butylhydroquinone (t-BHQ) were initially synthesized as a protective agent for a wide variety of chemical carcinogens [5]. This protective effect depends on the nuclear factor erythroid 2-related factor 2 (Nrf2)/antioxidant response element (ARE) pathway by increasing the activity of the second detoxification enzymes, including antioxidant enzymes such as malondialdehyde (MDA) and heme oxygenase 1 (HO-1) [6]. In addition, recently, the Phase II enzyme inducer was shown to bring neuroprotective effect against mitochondrial stress induced by mitochondrial complex II inhibitor 3-nitropropionic acid, by improving neurons survival, and attenuate the production of ROS in response to lipopolysaccharide treatment of cells [7]. However, there is no evidence to show these agents have a protective effect on diabetic cardiomyopathy.

MicroRNAs are endogenous, small noncoding RNAs, approximately 19–22 nucleotides in length, with complementary sequences in the 3′-untranslated regions (3′-UTRs) of downstream mRNAs to promote its degradation or inhibit translation process [8, 9]. Extensive studies have shown that microRNAs are involved in a wide variety of biological processes, including cell proliferation, differentiation, metastasis, apoptosis, and immune responses, also used as prognostic markers genes related to the development and progression of cardiovascular disease [10, 11]. For miR-503, some studies have showed that miR-503 was increased in diabetic muscles and oppositely correlated with cdc25 protein expression; in addition, miR-503 was also increased in myocardial microvascular endothelial cells from type 2 diabetic Goto-Kakizaki (GK) rats [12]; finally, compared with normal healthy controls, miR-503 was also increased in type 2 diabetes patients [13]. Therefore, miR-503 plays important role in diabetes; however, the role and mechanism of miR-503 in DCM remain unclear.

The aim of this study was to elucidate the essentially protective effect of Phase II enzyme inducer (CPDT) and the underlying mechanisms in diabetic cardiomyopathy. In this study, diabetic cardiomyopathy model in rats was duplicated, and Phase II enzyme inducer CPDT, as intervention agent, was used to detect relevant indicators, such as miR-503 and Nrf2/ARE signaling pathway, including Nrf2, HO-1 and MDA, to investigate whether the Phase II enzyme inducer (CPDT) reduces myocardial cell apoptosis, reduces the occurrence and development of diabetic cardiomyopathy through miR-503 and Nrf2/ARE signaling pathway, and provides new therapeutic targets for diabetic cardiomyopathy.

## 2. Material and Methods

### 2.1. Ethics Statement

The study was approved by the ethics committee of the Affiliated Hospital of Southwest Medical University, and all experimental procedures were approved by the animal care and use committee of the Affiliated Hospital of Southwest Medical University. The study was performed according to the recommendations of the guide for the care and use of laboratory animals.

### 2.2. Animals and Establishment of Diabetic Model and Treatment

The healthy male Wistar rats (180~220 g) were purchased from Southwest Medical University and housed at a temperature of 22 ± 1°C and humidity of 55 ± 5%. The rats were divided randomly into control group (C) and diabetes mellitus (DM) group. Rat diabetic model was established according to previous report [14] with slight modification. A high-fat diet was prepared with 20% lard, 5% cholesterol, 5% sucrose, 5% glucose, and 6% salt and emulsified in 20% Tween-80 and 30% propylene glycol with distilled water. Rats in the DM group were fed with 2 ml prepared high-fat diet per day. 30 mg/kg/d of streptozotocin (STZ) (Sigma, USA) in 0.1 M citrate buffer solution was injected intraperitoneally in diabetic rats for 3 consecutive days. 72 h later, fasting blood glucose levels were measured, and blood glucose levels > 16.7 mmol/l diabetes was regarded as diabetic model established successfully. The model rats were randomly divided into diabetic group and intervention group with 10 rats in each group. All the rats were fed with high sugar and high-fat diet, and the intervention group was given Phase II enzyme inducer (CPDT) (Sigma, St. Louis, MO, USA) 500 umol/kg/day for 14 days. All the rats were fed with high sugar and high-fat diet for 8 weeks.

### 2.3. Light and Electron Microscope Examination

For electron microscope examination, the heart was cut into small pieces and prefixed in 2.5% glutaraldehyde (0.2 M cacodylate buffer, pH 7.4) for 4 h, postfixed in 1% buffered sodium tetroxide for 1 h, and embedded in epoxy resin by routine methods. Ultrathin sections were examined using a JEM-1200 EX electron microscope (JEOL Ltd., Tokyo, Japan).

### 2.4. HE Staining

Tissues were fixed in 4% paraformaldehyde. The samples were embedded in paraffin, cut into 5 *μ*m thick sections and stained with hematoxylin-eosin (HE) for histological and collagen analysis, according to the manufacturers' protocols.

### 2.5. TUNEL Staining

Tissues were fixed in 4% paraformaldehyde tissue, and TUNEL fluorescence FITC kit (Roche, USA) was utilized for cell apoptosis assay as previous report [14]. Terminal deoxynucleotidyl transferase dUTP nick end labeling staining was used to detect DNA fragmentation (green fluorescence) with the nuclei stained with DAPI (blue fluorescence). Images were captured through fluorescence microscopy (Nikon Co., Japan).

### 2.6. RNA Extraction and qRT-PCR

Total RNA was extracted with the RNeasy plus mini kit (Qiagen, USA). Total RNA concentrations were determined by NanoVue plus (GE Healthcore, Piscataway, NJ, USA). miR-503 was detected with the TaqMan miRNA assays and U6 as an internal control. Nrf2, HO-1, and MDA were detected by qRT-PCR with the SYBR Premix Ex Taq II kit (TaKaRa, Dalian, China) and the Applied Biosystems ABI Prism 7500 HT sequence detection system. The primers of relative gene were as follows:

  Nrf2: 5′-ACGTGGCTAAGAATGTCATC-3′ (forward) and 5′-CTGGTAGGCGATGTCCTTA-3′ (reverse)
  HO-1: 5′-ACTCGAACGACTCTGATGATGT-3′ (forward) and 5′-GTCAGGTCTGCGAAACTTCTTA-3′ (reverse)
  MDA: 5′-TCTTCACAAATCCTCCCC-3′ (forward) and 5′-TGGATTAAAAGGACTTGG-3′ (reverse).

The expression of mRNA or microRNA was evaluated based on the threshold cycle (Ct) as *n* = 2^−ΔΔCt^, where ΔCt = Ct related mRNA − Ct reference and ΔΔCt = ΔCq  experimental − ΔCt control.

### 2.7. Western Blotting

The protein samples were collected and protein samples of 100 *μ*g each line were loaded on a 10% SDS-PAGE. Protein was transferred to PVDF membrane, which was subsequently blocked by 5% nonfat milk dissolved in PBS for 2 h; then, PVDF membrane was incubated with primary antibodies at 4°C overnight and then incubated with appropriate HRP-conjugated secondary antibodies at room temperature for 1 h. Finally, the bands were visualized with the chemiluminescence method and Western blotting bands were quantified using the Odyssey Infrared Imaging System (LI-COR, Lincoln, NE, USA). The Nrf2, HO-1, MDA, and *β*-actin antibody and HRP-conjugated secondary antibodies were purchased from Santa Cruz Biotechnology.

### 2.8. Prediction of Target Gene

Database of miRanda, TargetScan, and PicTar was used to predict the potential targets gene of miR-503. Among those target genes, nuclear factor erythroid 2-related factor 2 (Nrf2) had a binding site in the 3′-UTR of miR-503.

### 2.9. Luciferase Reporter Assays

The wild-type- (WT-) Nrf2 3′-UTRs and the mutated-Nrf2 3′-UTRs were synthesized by Sangon Biotech Co., Ltd. (Shanghai, China) and amplified by PCR. The forward primer was 5′-CCGCTCGAGAGGATCACTGAGGAAGGGGAAGTG-3′ and the reverse primer was 5′-ATAAGAATGCGGCCGCGCCTTGTACTACACATGTGTGACTGATC-3′.

The wild-type (WT) and mutated (Mut) exon of Nrf2 were inserted into the downstream of firefly luciferase reporter gene in the psiCHECK-2 vector. The constructed luciferase reporters were called psiCHECK-2-Nrf2-3′-UTR-WT and psiCHECK-2-Nrf2-3′-UTR-mut. For luciferase assay, The human embryonic kidney (HEK293T) 293 cells were seeded into 24-well plates, and NC mimics (empty vector) and miR-503 mimics (stable miR-503-overexpressing) were cotransfected with constructed reporter plasmids (0.2 ug) into HEK293T cells by Effectene transfection regents (Qiagen). After 48 hours, the luciferase activities were measured using the Clarity™ Luminescence Microplate Reader.

### 2.10. Primary Isolation and Culture of Myocardial Cells

Primary myocardial cells were from the hearts of 1–3-day-old Wistar rats. Briefly, cardiac tissues were separated and digested by pancreatin, the isolated cells were cultured at 37 °C, 5% CO2, and resuspended in Dulbecco's modified Eagle's medium (Hyclone, Logan, UT, USA) containing 10% fetal bovine serum (Hyclone); then, myocardial cells were purified through differential plating and 0.1 mM 5-bromo-2-deoxyuridine was used to deplete nonmyocardial cells.

### 2.11. Transfection of Relative MicroRNA

Synthetic miR-503 mimic and inhibitor were generated and purchased from Sangon Biotech Co., Ltd. (Shanghai, China), which had ability of upregulation and downregulation of miR-503. The mimic and inhibitor were transfected into myocardial cells with lipofectamine 2000 (Invitrogen, USA) to induce upregulation and downregulation of miR-503 in the myocardial cells according to manufacturer's instructions.

### 2.12. Statistical Analysis

Data were presented as mean values and standard deviation. The independent-samples* t-*test or the analysis of variance (ANOVA) was used to identify differences among all groups (SPSS, USA). The *P* value < 0.05 was considered to be statistically significance.

## 3. Results

### 3.1. Heart Parameters in Streptozotocin- (STZ-) Induced Diabetes and Treatment Rats

**Figure 1:**
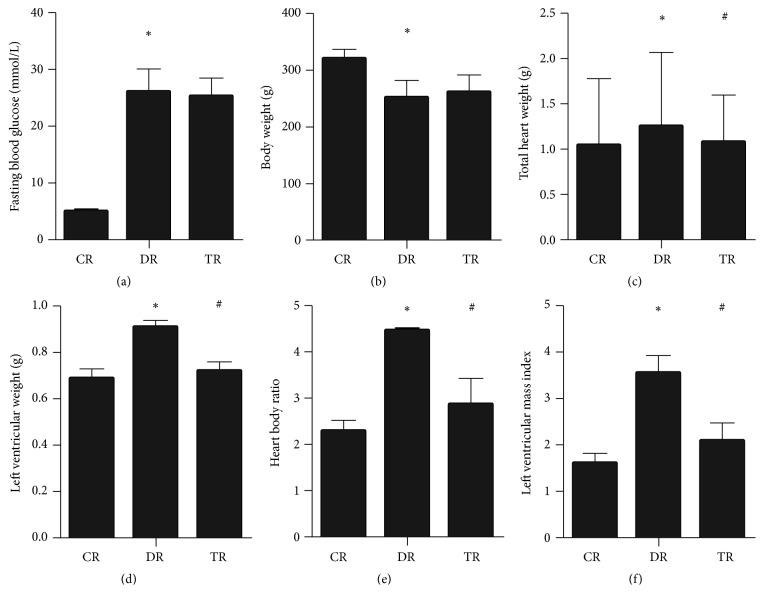
*Fasting blood glucose, body weight, and cardiac dysfunction in streptozotocin- (STZ-) induced diabetes and treatment rats;* CR: control rats; DR: diabetes rats; TR: treatment rats;** (**a-b) fasting blood glucose and body weight; (c–f) each weight's related indicators; *∗*: compared with control rats, *P* value < 0.05 was considered to be statistically significant; #: compared with diabetes rats, *P* value < 0.05 was considered to be statistically significant.

After the STZ was used to induce diabetes mellitus (DM), diabetic cardiomyopathy (DCM) was further established, DCM rats were treated with Phase II enzyme inducer (CPDT), and relevant information was detected and analyzed. The results showed that, compared with control rats, fasting blood glucose was significantly increased and body weight was significantly increased in diabetes and treatment rats, and there was no statistical difference in fasting blood glucose and body weight between diabetes and treatment rats (Figures 1(a) and 1(b)). In addition, compared with control rats, total heart weight, left ventricular weight, heart to body ratio, and left ventricular mass index were significantly increased in diabetes rats; however, compared with diabetes rats, these parameters were significantly improved in treatment rats (Figures 1(c)–1(f)).

### 3.2. Cardiopathology in Streptozotocin- (STZ-) Induced Diabetes and Treatment Rats

**Figure 2:**
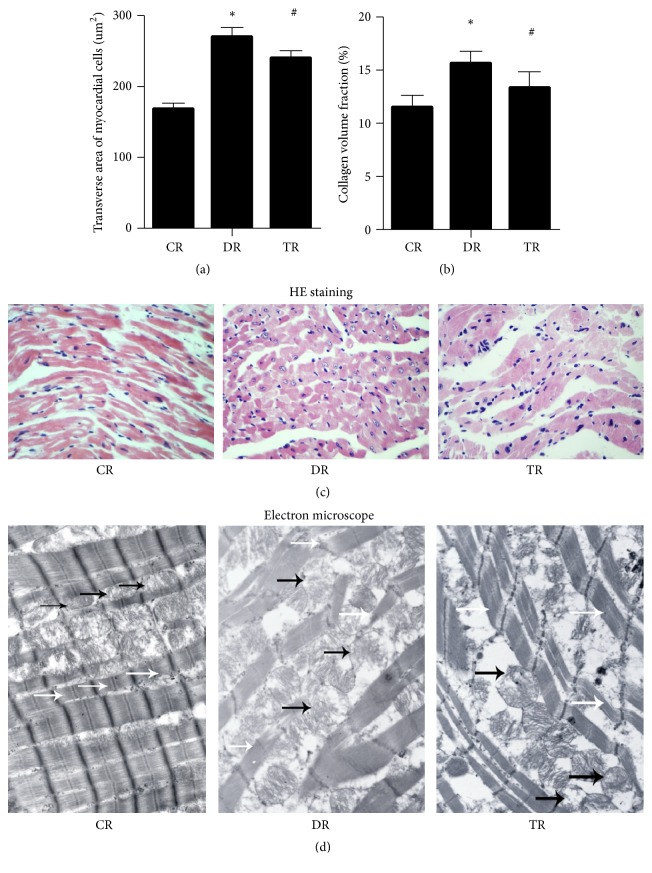
*Cardiopathology in streptozotocin- (STZ-) induced diabetes and treatment rats;* CR: control rats; DR: diabetes rats; TR: treatment rats. (a) Transverse area of myocardial cells; (b) collagen volume fraction OF Myocardial cell; (c) HE staining (×400); (d) electron microscope (×10000, the white arrow: cardiac fibers, and the black arrow: mitochondria); *∗*: compared with control rats, *P* value < 0.05 was considered to be statistically significant; #: compared with diabetes rats, *P* value < 0.05 was considered to be statistically significant.

**Figure 3:**
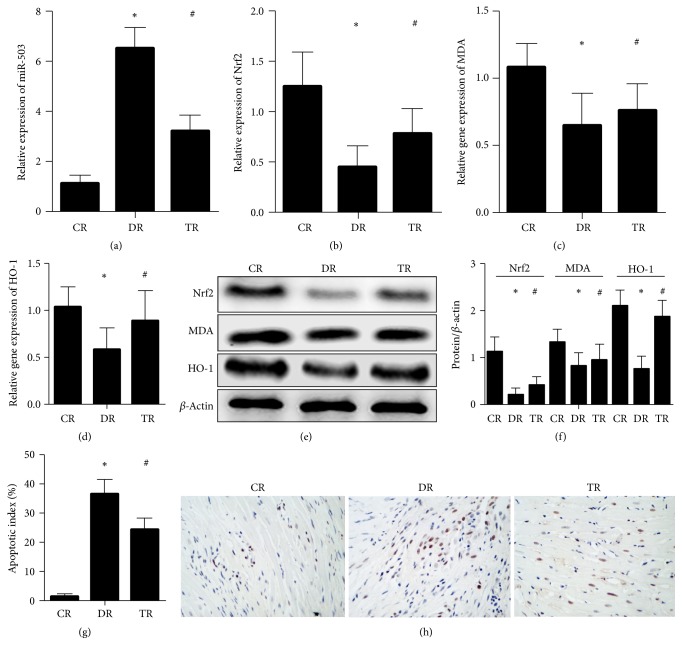
*The expression of miR-503, Nrf2, downstream medium, and apoptosis level in streptozotocin- (STZ-) induced diabetes and treatment rats;* CR: control rats; DR: diabetes rats; TR: treatment rats. (a) The expression of miR-503. (b) The gene expression of Nrf2. (c) The protein expression of Nrf2 and downstream medium. (d) The apoptosis level. (e) The apoptosis level by TUNEL staining (×200). *∗*: compared with control rats, *P* value < 0.05 was considered to be statistically significant; #: compared with diabetes rats, *P* value < 0.05 was considered to be statistically significant.

The animal model of diabetic cardiomyopathy was established, diabetic cardiomyopathy rats were treated with Phase II enzyme inducer (CPDT), and cardiopathology was detected and analyzed. The results from HE staining showed that, compared with control rats, myocardial hypertrophy and irregular arrangement myocardial cell were increased, myocardial cell nucleus was enlarged, the width of the intercellular space was widened, and the longitudinal connection between myocardial cells was decreased in diabetes rats; however, compared with diabetes rats, these parameters were significantly improved in treatment rats (Figures 2(a)–2(c)). The results from electron microscope examination showed that the cardiac fibers with regular arrays and the mitochondria were typically intact in control rats; however, the mitochondria and the myofibrils were swollen and disrupted in diabetes rats; however, these parameters were significantly improved in treatment rats (Figure 2(d)). The results from TUNEL staining showed that, compared with control rats, the apoptosis level was significantly increased in diabetes rats; however, compared with diabetes rats, the apoptosis level was significantly decreased in treatment rats (Figures 3(g) and 3(h)).

### 3.3. The Expression of miR-503, Nrf2, and Downstream Medium Level in Streptozotocin- (STZ-) Induced Diabetes and Treatment Rats

The diabetic cardiomyopathy was established, diabetic cardiomyopathy rats were treated with Phase II enzyme inducer (CPDT), and the miR-503, Nrf2, and downstream medium level were detected and analyzed. The result showed that, compared with control rats, miR-503 was significantly increased, and Nrf2, MDA, and HO-1 were significantly decreased in diabetes rats; however, compared with diabetes rats, miR-503 was significantly decreased, and Nrf2, MDA, and HO-1 were significantly increased in treatment rats (Figures 3(a)–3(f)).

### 3.4. The Nrf2 Was the Target Gene of miR-503

**Figure 4:**
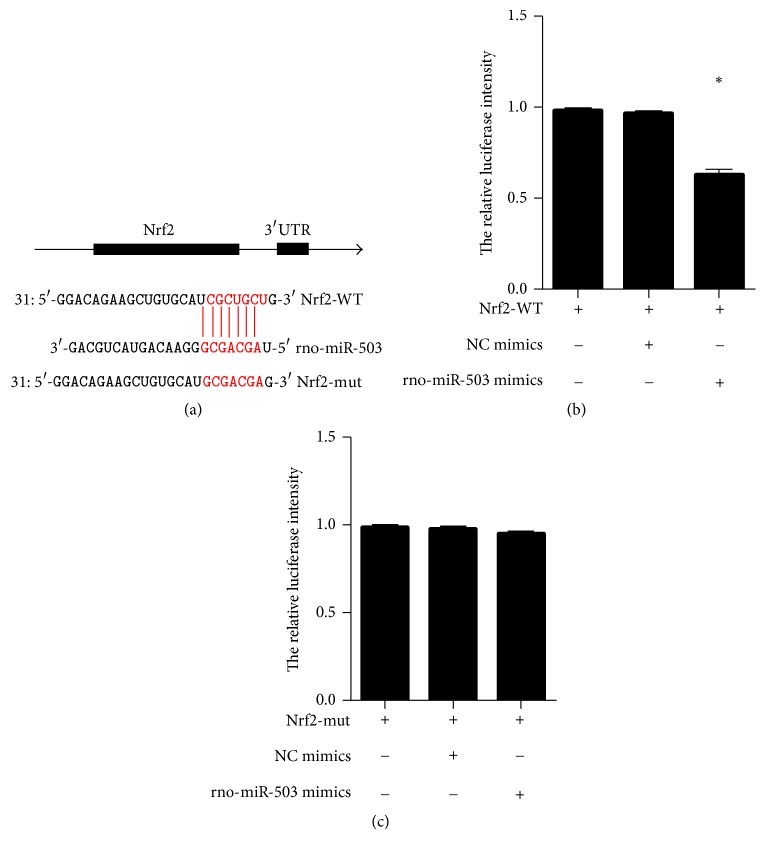
*The Nrf2 was the target gene of miR-503;* (a) putative miR-503 binding sequence in the wnt3a 3′-UTR and the site-directed mutant Nrf2 3′-UTR. (b, c) The WT or mut reporter plasmids or NC- mimics (empty vector) or rno-miR-503 mimics (stable miR-503-overexpressing) were cotransfected into HEK293T cells. Relative repression of firefly luciferase expression was standardized to a transfection control. ^*∗*^*P* < 0.05.

According to the prediction analysis of the TargetScan, PicTar, and miRanda data, Nrf2 has a putative binding site in the 3′-UTR of miR-503 and was speculated as the target gene of miR-503 (Figure 4(a)). The dual-luciferase reporter assay method was used to further investigate whether miR-503 directly targets Nrf2. The results showed that miR-503 suppressed luciferase activity through 3′-UTR of wild-type (WT) HMGB1 (Figure 4(b)). In addition, the activity change of fluorescence of miR-503 containing the mutated 3′-UTR site or the control group lacking an Nrf2 3′-UTR sequence had no statistically significant difference (Figure 4(c)).

### 3.5. miR-503 Regulated the Expression of Nrf2 in Myocardial Cells

**Figure 5:**
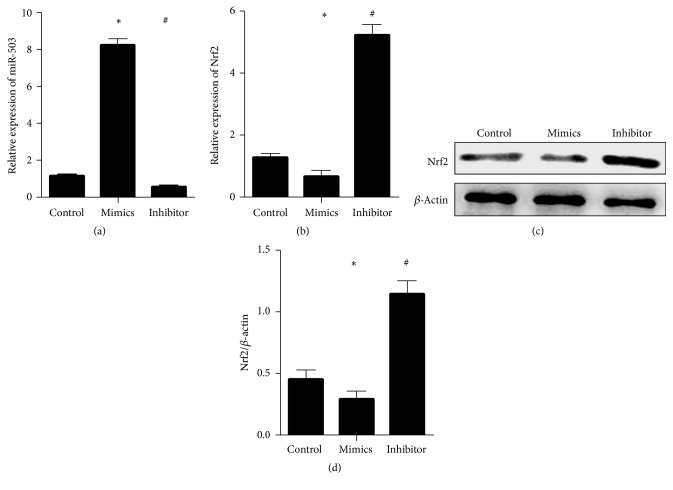
*miR-503 regulated the expression of Nrf2 in myocardial cells;* (a) the expression of miR-503; (b) the gene expression of Nrf2; (c-d) the protein expression of Nrf2; *∗*: compared with control, *P* value < 0.05 was considered to be statistically significant; #: compared with mimics, *P* value < 0.05 was considered to be statistically significant.

After mimic and inhibitor were transfected into myocardial cells, the miR-503, Nrf2, and downstream medium level were detected and analyzed. The results showed that, compared with control, the intracellular miR-503 was increased by miR-503 mimics and decreased by miR-503 inhibitor (Figure 5(a)). In addition, the expression of Nrf2 was decreased by miR-503 mimics and increased by inhibitor (Figures 5(b)–5(d)).

## 4. Discussion

Diabetic cardiomyopathy (DCM) is a common structural abnormality caused by myocardial cell injury in diabetes, leading to left ventricular hypertrophy and diastolic or systolic dysfunction [15]. Recently, numerous studies have reported that DCM is a kind of independent pathophysiology, and the main clinical manifestations are congestive heart failure due to cardiac diastolic and systolic dysfunction [16, 17]. Nowadays, the pathological mechanism of this disease still remains unclear. In addition, treatment for this disease is not ideal and it is difficult to prevent and treat this disease, ultimately leading to heart failure, arrhythmia, and cardiogenic shock, one of the important causes of sudden death [18]. In this study, we found that miR-503 is involved in the progress of DCM, and Phase II enzyme inducer (CPDT) could reverse the impaired structure and function, reduce myocardial apoptosis, relieve the occurrence and development of DCM through miR-503 and Nrf2/ARE signaling pathway, and provide new therapeutic targets for DCM.

Some studies have showed that the changes of tissue structure and function, oxidative stress, myocardial apoptosis, and necrosis are the important factors leading to occurrence and development of DCM [19, 20]. In the early DCM, myocardial apoptosis and necrosis occurred, oxidative stress was increased, and the corresponding signal transduction pathway and gene expression were abnormal; thereby the programmed cell death was initiated [21]. In addition, some studies have also showed that in the development stage of DCM, during high blood glucose, the collagen glycosylation, degradation, and endocardial collagen deposition happened, the death of myocardial cells gradually is replaced, eventually, the ventricular remodeling appeared, in addition, in this process, the renin angiotensin aldosterone system (RAS) is activated, the cytokines is increased, and the fibroblast proliferation and myocardial fibrosis are promoted [22, 23]. In this study, we found that myocardial structure and function were impaired and myocardial cell apoptosis level was increased in diabetes rats and these results were consistent with previous studies; model of DCM was successful. In addition, the impaired structure and function were reversed and the myocardial cell apoptosis level was decreased in diabetes rats after CPDT treatment. For CPDT, it has been reported to have a protective potential in neurogenic bladder cells and tissues [24–26]. In this study, for DCM, we also found that the CPDT had a protective potential; however, this mechanism remains unclear.

MicroRNAs have been reported to be involved in the pathogenesis of common human diseases, such as the nervous system diseases, cardiovascular system diseases, and cancer. Moreover, microRNAs have been reported to participate in the important physiological functions, such as autophagy, lysosome, autolysis, apoptosis, and cell cycle regulation [27]. In addition, microRNAs have also been reported to have ability of treating many diseases and microRNAs are potential to be a target for disease treatment [28]. Many studies have demonstrated that many abnormal microRNAs were involved in numerous important pathophysiological processes in DCM, such as myocardial hypertrophy, myocardial fibrosis, myocardial apoptosis, mitochondrial dysfunction, myocardial electrical remodeling, and epigenetic modification [29, 30]. In this study, we found that miR-503 was also increased in DCM and closely correlated with occurrence and development of DCM. In addition, exhilaratingly, we also found that miR-503 was shown to be decreased in DCM treated by CPDT. Therefore, miR-503 was involved in DCM and CPDT exerted myocardial protection by performing miR-503.

To further investigate the downstream molecular mechanism of miR-503 in myocardial protection, the prediction analysis and dual-luciferase reporter assay method were used, and the results showed that nuclear factor erythroid 2-related factor 2 (Nrf2) had a putative miR-503-binding site mapped to the 3′-UTR and Nrf2 was the target gene of miR-503. Nrf2 is a novel transcription factor that belongs to the transcription factor Cap'n' Collar family, associated with the cytoplasmic chaperone Keap1 in physiological state and plays important role in the regulation of oxidative stress response [31]. Under the source of oxidative stress, Nrf2 and Keap1 solution is transferred into cell nucleus, combined with the antioxidant response element ARE locus, and the ARE was activated, playing an important role in protecting cells [32]. Therefore, Nrf2/ARE is one of the most important antioxidant pathways, playing an important role in the field of resistance to oxidation and chemical stimulation. Accumulating evidence has showed that a lot of oxygen free radicals were produced by oxidative stress in myocardial tissue, which can directly or indirectly damage the myocardium or affect the myocardial systolic and diastolic function and participate in the development of diabetic cardiomyopathy [33]. In addition, many oxygen free radical formations mediated myocardial cell apoptosis and also participate in the development of DCM [34]. In this study, we found that Nrf2 was decreased, myocardial apoptosis was increased in diabetes rats, and Phase II enzyme inducer (CPDT) had ability of upregulating expression of Nrf2 and reducing myocardial apoptosis in DCM. For CPDT, some studies have also showed that the protective function could depend on Nrf2/ARE pathway and CPDT could regulate the expression of Nrf2. Therefore, miR-503 is involved in the progress of DCM via regulating Nrf2/ARE signaling pathway, CPDT reduces the development of DCM through miR-503 and Nrf2/ARE signaling pathway. In development of DCM, miR-503 was increased, Nrf2 was decreased, antioxidative stress ability was weakened, and myocardial apoptosis was increased, damaging the myocardium or affecting the myocardial systolic and diastolic function. In treatment of CPDT for DCM, miR-503 was decreased, Nrf2 was increased, antioxidative stress ability was improved, and myocardial cell apoptosis was decreased, protecting the myocardium and improving the myocardial systolic and diastolic function.

In this study, we also investigated the downstream medium of Nrf2 in development of DCM and treatment of CPDT for DCM. We found that, in this progress, when Nrf2 was activated, malondialdehyde (MDA) and heme oxygenase 1 (HO-1) were also activated, and when Nrf2 was inhibited, MDA and HO-1 were also inhibited. The MDA is the final product of lipid peroxidation reaction of oxygen free radicals, attacks the unsaturated fatty acids, and plays an important role in the process of oxidative stress damage [35]. The HO-1 is a kind of microsomal oxidase with multiple functions and plays an important role in the process of oxidative stress [36]. These results also further confirmed that oxygen free radical attack occurs in DCM, and CPDT can reduce the content of oxygen free radicals and protect the myocardium.

In conclusion, miR-503 was involved in the progress of DCM via regulating Nrf2/ARE signaling pathway, and the CPDT reduces the occurrence and development of diabetic cardiomyopathy through miR-503 and Nrf2/ARE signaling pathway.
